# The Strength-Interval Curve in Cardiac Tissue

**DOI:** 10.1155/2013/134163

**Published:** 2013-02-20

**Authors:** Sunil M. Kandel, Bradley J. Roth

**Affiliations:** Department of Physics, Oakland University, Rochester, MI 48309, USA

## Abstract

The bidomain model describes the electrical properties of cardiac tissue and is often used to simulate the response of the heart to an electric shock. The strength-interval curve summarizes how refractory tissue is excited. This paper analyzes calculations of the strength-interval curve when a stimulus is applied through a unipolar electrode. In particular, the bidomain model is used to clarify why the cathodal and anodal strength-interval curves are different, and what the mechanism of the “dip” in the anodal strength-interval curve is.

## 1. Introduction

Despite the wide use of defibrillators, the mechanism of defibrillation is poorly understood. After decades of continuous research, millions of dollars have been invested, and thousands of research papers have been published. Yet, the question of “how does an electric shock delivered by a defibrillator interact with cardiac tissue to terminate ventricular fibrillation?” remains unanswered. Our understanding of how a defibrillator influences cardiac tissue is still developing and many important questions need to be resolved. Defibrillation of the heart is a complex process, which we cannot hope to understand until we have an adequate description of the more simple process of electrical stimulation of cardiac tissue by unipolar electrode. Therefore, this paper focuses on unipolar stimulation.

The basic mechanism of defibrillation is still not clear [1–3]. One leading hypothesis is virtual electrode polarization (VEP), that is, the simultaneous presence of depolarized and hyperpolarized regions around a stimulation site. It is believed that VEP plays an important role in electrical stimulation of cardiac tissue [4–6]. To appreciate the importance of VEP, one needs to understand the shape of the strength-interval (SI) curve completely. The strength-interval curve indicates the tissue's excitability. It measures the threshold stimulus strength necessary to generate an action potential at different time intervals after a previous action potential has been generated. The goal of this paper is to survey several studies, most based on mathematical modeling, of the cardiac strength-interval curve.

## 2. Virtual Electrodes

**Figure 1:**
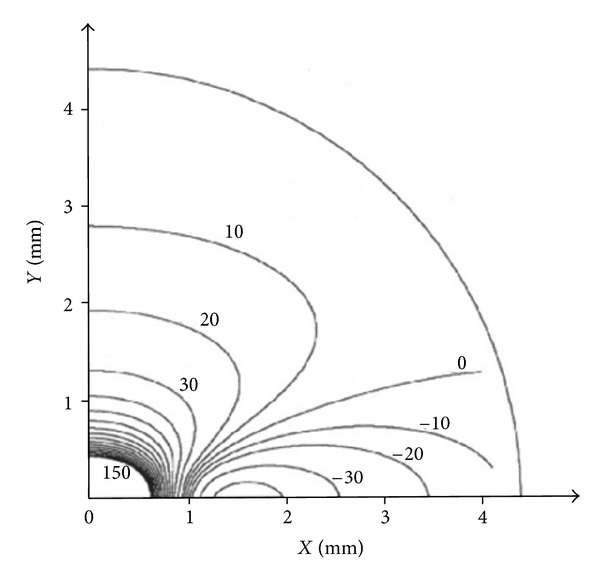
The isopotential contours for the transmembrane potential induced in a two-dimensional sheet of tissue by a unipolar extracellular electrode, located at the origin. Only one quadrant of the sheet is shown. The fibers are along the *x*-axis. Contours are drawn every 10 mV. The contours near the electrode are closely spaced and are not shown [7].

In 1989, Sepulveda et al. [7] calculated the steady-state response of a passive two-dimensional sheet of cardiac tissue to a unipolar stimulus using the bidomain model, which accounts for the anisotropy of both the intracellular and extracellular spaces. When a tissue with unequal anisotropy ratios in the two spaces is excited with a point current source, it induces a complicated spatial distribution of transmembrane potential that includes adjacent regions of depolarized and hyperpolarized tissues. The induced region of depolarization is called the virtual cathode and the region of hyperpolarization is called the virtual anode. Sepulveda et al. [7] calculated the locations of virtual anodes and cathodes around a unipolar electrode in anisotropic cardiac tissue and found strong depolarization under the cathodal electrode, and adjacent hyperpolarization near the electrode along the fiber direction (Figure 1). This predicted distribution of polarization was observed experimentally six years later [4, 8, 9]. 

**Figure 2:**
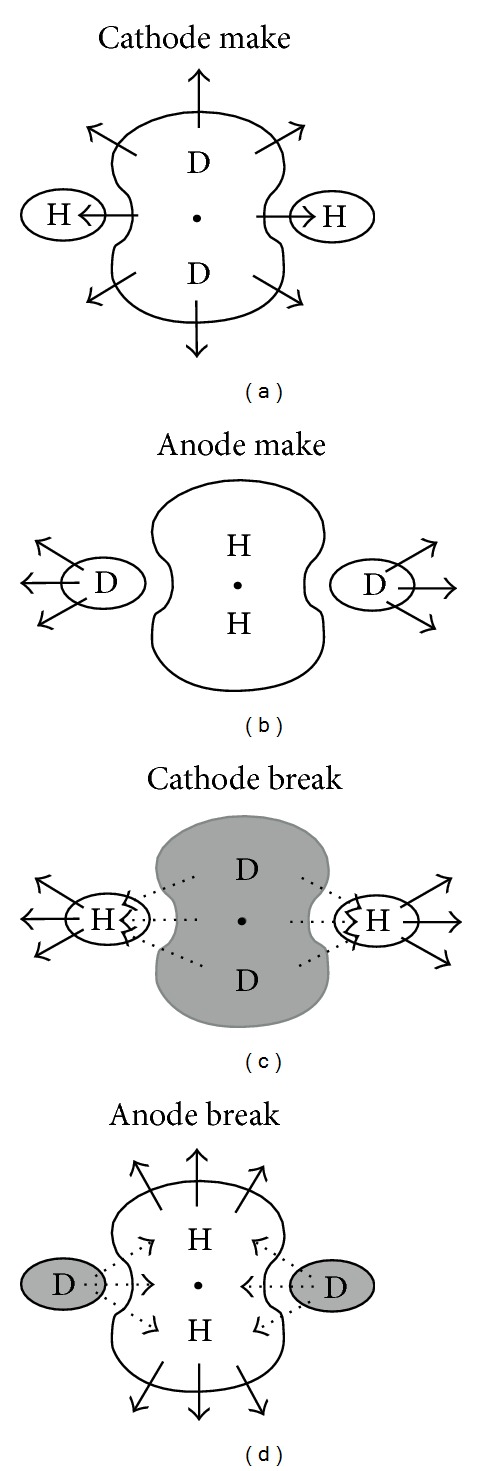
A schematic illustration of the four mechanisms of excitation (a) cathode make; (b) anode make; (c) cathode break; (d) anode break. Fiber orientation is along the horizontal and the electrode is indicated by the black dot. D is depolarization and H is hyperpolarization. This figure summarizes the results of simulations performed using the bidomain model [10].

Dekker [11] identified four mechanisms of excitation in the heart: cathode make, cathode break, anode make, and anode break. Roth [5] used an active version of the bidomain model to explain these four mechanisms, including anode break excitation (Figure 2). Wikswo et al. [4] verified these mechanisms by using optical mapping [12] in rabbit hearts.

Make excitation occurs after the stimulus is turned on. There are two types of make excitation: cathode make and anode make. In cathode make excitation, the tissue under the electrode is depolarized, exciting an action potential wave front (Figure 2(a)). At large stimulus strengths cathode make stimulation becomes more complex, producing a “dog-bone-” shaped region of excitation as shown experimentally by Wikswo et al. [13] and simulated by Roth and Wikswo [14]. Cathode make excitation is the mechanism associated with pacing. 

In anode make excitation, a strong anodal stimulus is applied that hyperpolarizes the tissue directly below the electrode but depolarizes the tissue at two virtual cathodes one or two millimeters from the anode along the fiber direction (Figure 2(b)). A strong stimulus is necessary to produce enough depolarization at the virtual cathodes to excite propagating wave fronts, and therefore anode make excitation has a higher threshold than cathode make excitation. The condition of unequal anisotropy ratios is necessary for anode make excitation, but not for cathode make excitation. 

Break excitation occurs after the stimulus is turned off and plays an important role in stimulating refractory tissue. There are two types of break excitation: cathode break and anode break. In cathode break excitation, a strong cathodal stimulus is applied when the tissue is refractory from the previous action potential. After the stimulus is applied, the depolarization under the electrode cannot excite an action potential if the tissue is refractory (unexcitable), but the hyperpolarization at the virtual anodes causes the tissue there to recover from the refractoriness quickly. After the stimulus is turned off, the depolarization under the cathode diffuses into the adjacent hyperpolarized region, exciting the tissue and initiating wave fronts that propagate along the fiber direction (Figure 2(c)). Cathode break excitation has a higher threshold than cathode make excitation because this mechanism requires that the hyperpolarization at the virtual anodes on each side of the cathode is strong enough to de-excite the tissue (force it to recover from refractoriness). 

**Figure 3:**
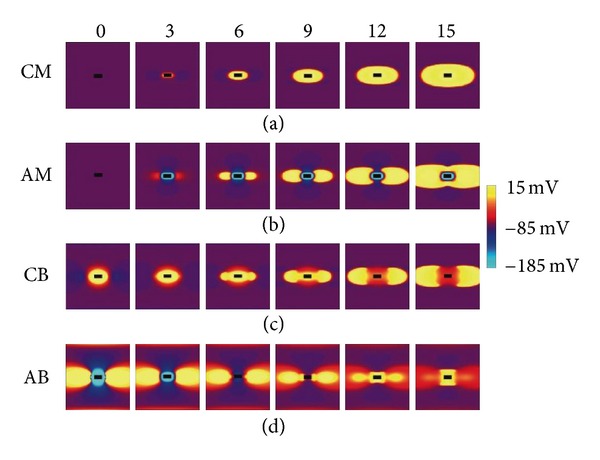
The calculated transmembrane potential during or following unipolar stimulation of cardiac tissue. The four rows correspond to the four mechanisms of stimulation: cathode make (CM), anode make (AM), cathode break (CB), and anode break (AB). The fibers are horizontal, and the transmembrane potential is shown by color. An 8 mm by 8 mm region of tissue is shown, and the black rectangle at the center of each region is the position of the electrode. The numbers along the top of the frame are the times in milliseconds [15].

In anode break excitation, a strong anodal stimulus is applied when the tissue is refractory from the previous action potential. As soon as the stimulus is applied, the tissue under the anode is strongly hyperpolarized and made excitable, but the tissue at the virtual cathodes is depolarized and remains unexcitable. After the stimulus is turned off, depolarization at the virtual cathodes diffuses into the hyperpolarized tissue under the anode, exciting wave fronts that propagate perpendicular to the fiber direction (Figure 2(d)). Anode break stimulation behaves similar to cathode break stimulation, occurring by diffusion of depolarization into excitable tissue. The depolarization at the virtual cathode diffuses into a strongly hyperpolarized region under the anode (and not vice versa) because the depolarization decays more slowly than the hyperpolarization. Anode break excitation has the highest threshold of the four mechanisms because a strong stimulus is necessary to create enough depolarization to diffuse into excitable tissue and initiate a wave front.  Figure 3 shows the transmembrane potential distribution at several times following cathode make, anode make, cathode break, and anode break excitation.

## 3. Strength-Interval Curve

The strength-interval curve describes how refractory cardiac tissue responds to an electrical stimulus. It is measured by applying two stimuli—a first (S1) that initiates an action potential, and then a second (S2)—and plotting the S2 threshold strength as a function of the S1-S2 time interval. The shape of the strength-interval curve depends on the polarity of the stimulus. 

Dekker [11] measured the strength-interval curve for all four mechanisms of excitation in dog hearts. His make curves decreased monotonically with the interval, while his break curves contained a dip, in which the S2 threshold increases as the interval increases. He also found that at long intervals, cathode make excitation has the lowest stimulus threshold, followed by anode make, cathode break, and finally anode break.

If a cathodal stimulus is applied through a unipolar electrode, the strength-interval curve is easy to understand. It decreases monotonically as the interval increases, because the tissue becomes easier to stimulate as it recovers from the refractoriness caused by the S1 action potential. If an anodal S2 stimulus is applied through the same electrode, however, the response becomes more complicated. A section of the curve has positive slope: threshold increases as the interval increases [11, 16–20], thereby paradoxically making the tissue harder to stimulate as it recovers from refractoriness. This behavior is known as the “dip” in the anodal SI curve. 

 The dip found in the anodal strength-interval curve raises two questions.

1. What is the mechanism of the dip?
2. Why does a dip appear in the anodal SI curve but not in the cathodal SI curve?

These questions remained unanswered until the development of the bidomain model of cardiac tissue.

The bidomain model provides the theoretical understanding of electrical excitation of the heart. It is a two- or three-dimensional cable model that accounts for the anisotropy of the intracellular and extracellular spaces. The electrical behavior of the tissue is governed by a pair of coupled partial differential equations. The model was developed in the late 1970s [21–23]. Calculations by Plonsey and Barr [24, 25] in the mid 1980s and by several researchers in the late 1980s [26–28] established the model as an important tool for simulating the electrical behavior of cardiac tissue. Sepulveda et al.'s [7] calculation of the transmembrane potential during electrical stimulation (Figure 1) introduced the use of the model for analyzing pacing and defibrillation. The review by Henriquez [29] remains a useful overview of the bidomain model. The bidomain model has resulted in a series of theoretical predictions that have been confirmed experimentally, usually using optical mapping [6]. Bidomain simulations have clarified the mechanism of make and break excitation, and the dip in the anodal strength-interval curve. 

**Figure 4:**
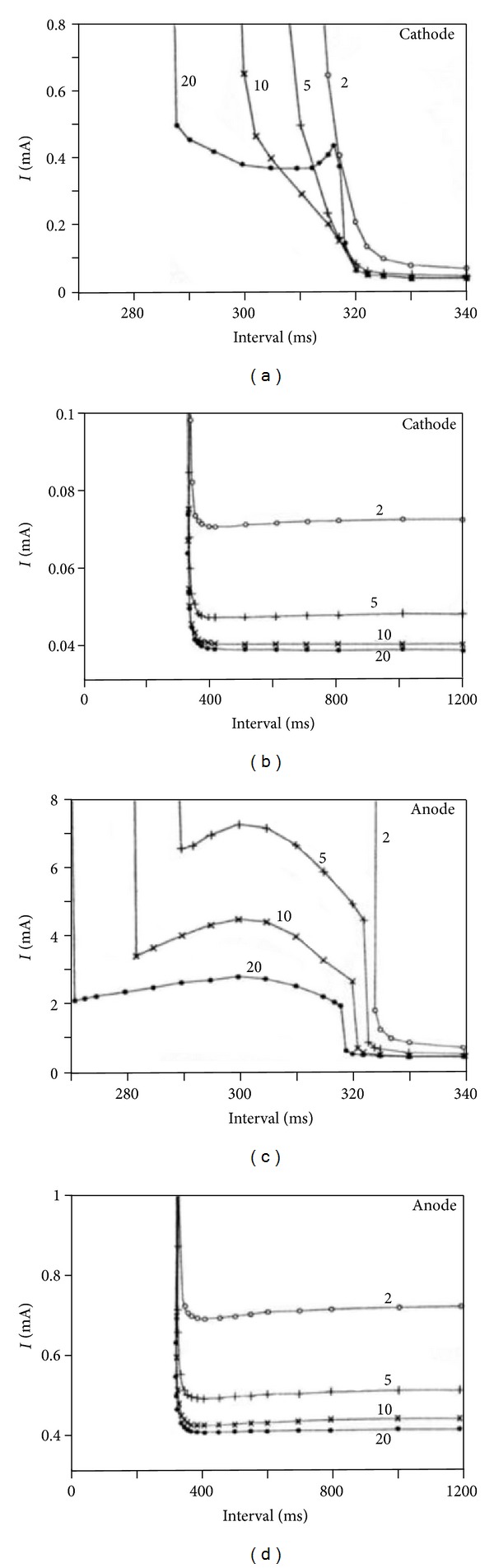
The calculated cathodal (a) and anodal (c) strength-interval curves for S2 pulse durations of 2, 5, 10, and 20 msec. (b) and (d) are the same cathodal and anodal strength-interval curves at long intervals [10].

Figure 4 shows the cathodal and anodal strength-interval curves for different S2 pulse durations calculated using the bidomain model [10]. The cathodal SI curve shows the initial decrease in the threshold stimulus strength (S2) as the interval increases (Figure 4(a)). At very long intervals, the slope of the strength-interval curve becomes slightly positive and recovers to resting threshold very slowly (Figure 4(b)), but at most the strength-interval curve is 3% below the diastolic threshold due to the slow recovery of  *V*_*m*_ to its resting value. This behavior is intrinsic to the model used to represent the ion channel kinetics [31] and does not depend on the bidomain properties of the tissue.

In Figure 4(a), the 20 ms cathodal curve falls abruptly at about 318 ms. The wave front dynamics during and following this S2 interval show that 318 ms corresponds to the transition from cathode make to cathode break excitation [10, 32]. The positive slope between 310 and 318 ms is due to the interaction between the cathode make and cathode break mechanisms of stimulation. Shorter duration (2, 5, and 10 ms) S2 stimuli do not contain any region of positive slope and the cathodal SI curves are monotonically decreasing. 

Anodal strength-interval curves are more complex than cathodal curves.  Figure 4(c) shows the predicted anodal strength-interval curves for four different stimulus pulse durations. Each curve contains an abrupt fall (a discontinuity) at about 320 ms and is divided into two sections, corresponding to break excitation for intervals less than 320 ms, and to make excitation for intervals greater than 320 ms. The transition is more distinct for anodal than cathodal excitation because of the different directions of propagation following anode break and anode make. In anode make excitation, the propagation occurs in the direction parallel to the myocardial fibers, whereas in anode break excitation, the propagation occurs in the direction perpendicular to the fibers. Furthermore, the make section is monotonically decreasing, but the break section contains a dramatic region of positive slope, corresponding to the dip. 

**Figure 5:**
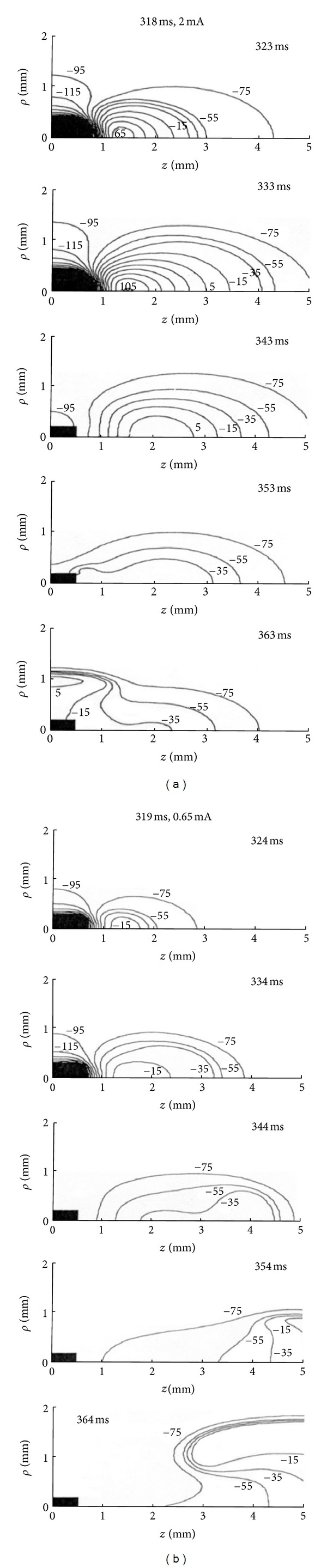
Contour plots of transmembrane potential as a function of *z* (parallel to the fibers) and *ρ* (perpendicular to the fibers) during and following a 20 ms duration anodal S2 stimulus. In (a), S2 has a threshold strength of 2.0 mA and is applied at 318 ms. In (b), S2 has a threshold strength of 0.65 mA and is applied at 319 ms. Only one quadrant of the *z*-*ρ* plane is shown. The black rectangular box is the electrode [10].

To obtain a more detailed understanding of the discontinuity in the anodal SI curve, consider the wave front dynamics during and following a threshold stimulus strength S2. When an anodal stimulus of 2 mA is applied at 318 ms for 20 ms duration (Figure 5(a)), depolarization at the virtual cathode initiates a very weak wave front. Since the tissue is refractory, the wave front cannot propagate further and dies out. After the pulse ends, the depolarization at the virtual cathode interacts electrotonically with the hyperpolarized anode and triggers a wave front by the anode break mechanism. As a result the wave front is propagated in the direction perpendicular to the fibers. Figure 5(b) shows the wave front dynamics if a smaller stimulus of 0.65 mA is applied at 319 ms for 20 ms duration (only 1 ms later than in Figure 5(a)). In this case, depolarization at the virtual cathode initiates a wave front that can propagate outward in the direction of the fibers by anode make excitation. 

In Figure 4(c), for intervals less than 300 ms, the anode-break threshold increases with increasing interval. To understand why this dip is present, realize that anode break excitation requires a source of depolarization that interacts electrotonically with the hyperpolarized and excitable tissue under the anode. This depolarization can arise from two sources. One is the depolarization caused by the S2 stimulus (the virtual cathodes), and another is the surrounding depolarization from the previous S1 action potential. If S2 is applied during the repolarization phase of the S1 action potential, then making the S1-S2 interval shorter means that there is more depolarization present from the S1 action potential, reducing the S2 threshold. Thus, the dip in the anodal strength-interval curve arises from the electrotonic interaction of the hyperpolarized tissue under the anode and the adjacent depolarized tissue. Other reasons for the dip in the anodal strength-interval curve are the lack of an active response at the virtual cathode and the decrease in the membrane resistance with time during the dip reflecting the repolarization of the action potential and the inward rectification of the cardiac membrane [10]. 

The anode break mechanism can occur for intervals down to a minimum value. (In Figure 4(c), this minimum is 280 ms for the 10 ms S2 duration SI curve.) If S2 is applied earlier than the minimum value, a wave front is initiated by the anode break mechanism, but when this wave front propagates several millimeters from the anode it encounters refractory tissue and dies. In this case, no excitation is said to occur and the S2 curve rises to infinity.

## 4. Modifications to the Strength-Interval Curve

The mechanism for the dip in the anodal strength-interval curve implies that the dip should occur during the repolarization phase of the S1 action potential. One way to test this prediction is to make the repolarization phase of the previous action potential occur at a different time than the recovery from refractoriness. This is possible in cases of postrepolarization refractoriness, when the action potential remains refractory long after the previous action potential has returned to rest [35].

**Figure 6:**
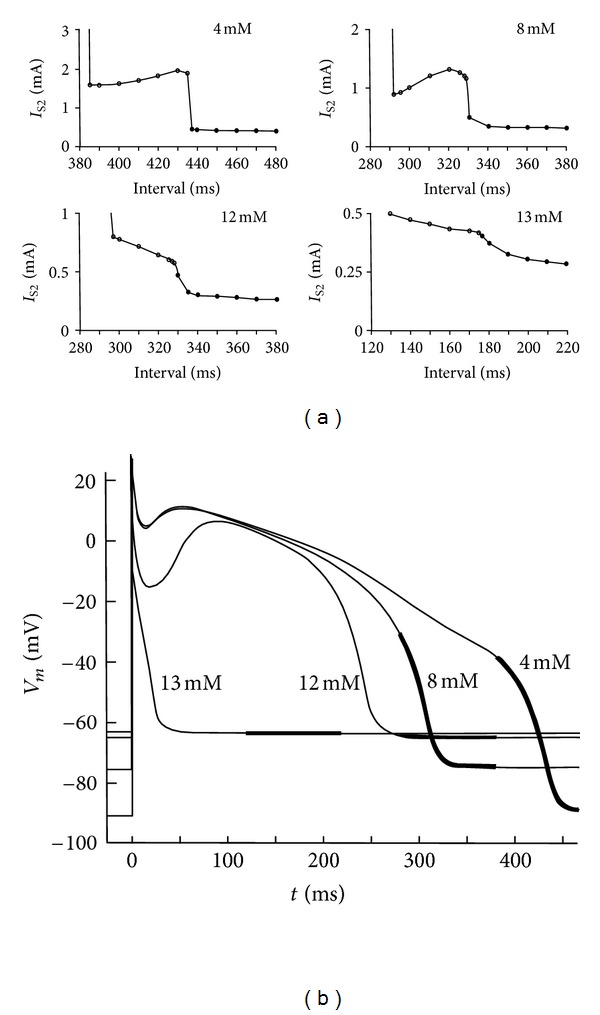
(a) The calculated anodal strength-interval curve for an S2 pulse duration of 20 ms and extracellular potassium concentrations of 4, 8, 12, and 13 mM. Open circles indicate break excitation and filled circles indicate the make excitation. (b) The transmembrane potential as a function of time for extracellular potassium concentrations of 4, 8, 12, and 13 mM. The thick section of each curve indicates the time for which the strength-interval curves are calculated in (a) [30].

Roth and Patel [30] examined the anodal strength-interval curve for varying extracellular potassium concentrations. High extracellular potassium favors break over make excitation.  Figure 6 shows the anodal strength-interval curves for four extracellular potassium concentrations. At high potassium, the dip in the anodal strength-interval curve disappears, and the transition from make to break excitation is less abrupt. The break threshold can become so low that break excitation is responsible for exciting the resting tissue. For the simulations of Figure 6, this occurs at a potassium concentration of 13.3 mM [30]. High potassium raises the resting potential, depolarizing the tissue. As already discussed, anode break excitation requires a source of depolarization that interacts electrotonically with the hyperpolarized and excitable tissue under the anode. The depolarized resting potential supplies this additional depolarization, making break excitation easier. Patel and Roth found that the reason for the disappearance of the dip in the anodal strength-interval curve was that the tissue showed postrepolarization refractoriness: the tissue remained refractory after the transmembrane potential returned to its resting state. In that case, as the interval decreased the refractoriness increased, but this did not coincide with an increase in the surrounding depolarization caused by the S1 action potential. Sidorov et al. [37] studied hyperkalemia using optical mapping and found that the diastolic anodal excitation mechanism changed from make (4 mM KCl) to break (8 mM KCl). They also found that at high potassium, the dip in the break section of the anodal strength-interval curve disappears.

**Figure 7:**
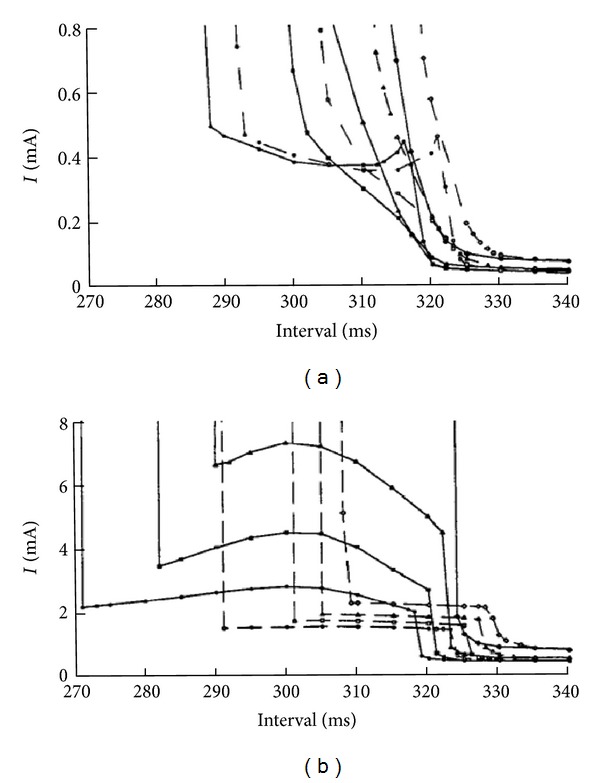
Cathodal (a) and anodal (b) strength-interval curves calculated without (solid) and with (dashed) electroporation, for S2 pulse duration of 20, 10, 5, and 2 ms [33].

Bray and Roth [33] studied the effect of electroporation on unipolar stimulation and the strength-interval curve in cardiac tissue. They used the bidomain model with unequal anisotropy ratios to represent the tissue and the Beeler-Reuter model [31] extended to include a simple model of electroporation [39] to represent the ion channel kinetics of the membrane. They found that for cathodal stimulation electroporation does not dramatically affect the threshold for a cathode make stimulus but it shifts all the cathode break strength-interval curves about 5 ms towards the right (Figure 7(a)). For anodal stimulation, they found out that electroporation lowers the anode break threshold and also shifts the entire anode make and anode break strength-interval curves to the right (Figure 7(b)). 

Electroporation also reduces or eliminates the “dip” in the anodal strength-interval curve because it supplies another source of depolarization following the end of an anodal current pulse—the short circuiting of the membrane toward zero potential—which adds to the diffusion of depolarization from the nearby virtual cathodes, but which does not decrease as time increases. They also simulated the tissue with equal anisotropy ratios and found that electroporation provides a new mechanism for anode break stimulation that is only dependent on the membrane behavior.

**Figure 8:**
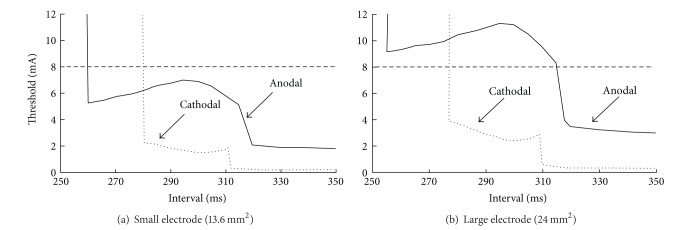
The cathodal and anodal strength-interval curves calculated using 20 ms S2 pulse duration for a small electrode in (a) and a large electrode in (b). The dashed line indicates the peak output of an electrical stimulator (8 mA). For the small electrode, both the anodal and cathodal curves lie below 8 mA, and for the large electrode, the cathodal curve lies below 8 mA but the break section of anodal curve lies above 8 mA [34].

Mehra et al. [40] implanted a pacing electrode in a dog heart and observed that the anodal refractory period is shorter than the cathodal refractory period immediately after the implantation. But after a few weeks, the cathodal refractory period becomes shorter than the anodal refractory period. Bennett and Roth [34] examined this experiment using numerical simulations based on the bidomain model and the Beeler-Reuter model for the membrane kinetics [31]. They assumed that when a pacing electrode is first implanted, the tissue adjacent to the electrode is healthy and excitable. After several weeks, however, unexcitable scar tissue forms around the electrode, making its effective size larger. For smaller electrodes both anodal and cathodal threshold stimuli are low, but for larger electrodes the anodal threshold stimulus is so large that the stimulator Mehra et al. used could not provide enough current to trigger anode break excitation (Figure 8). In that case, the break section of anodal strength-interval curve could not be observed and experimentalists would define the time of abrupt discontinuity associated with make and break transition as the end of refractory period. If they had used a more powerful stimulator, they would have uncovered the anode break section of the strength-interval curve and found that the true end of the refractory period was much earlier. Bennett and Roth [34] concluded that the anodal refractory period changes little with time, and Mehra et al.'s [40] observation was just an artifact caused by the output limit of the stimulator they used. 

**Figure 9:**
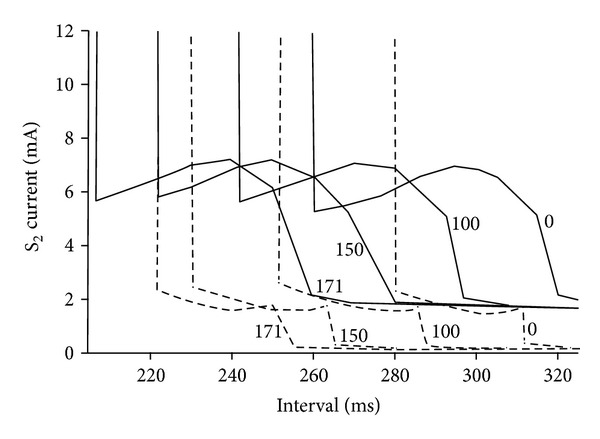
The calculated anodal (solid line) and cathodal (dashed line) strength-interval curves for the pulse duration of 20 ms. S1 pacing rate was 0, 100, 150, and 171 beats/min. [36].

Rapid pacing induces fibrillation in the heart. Bennett and Roth [36] studied how the pacing rate affects the strength-interval curve in cardiac tissue. For all SI pacing rates, they found make and break sections in each curve and a prominent dip in the break section of anodal curve and concluded that the overall shape of the strength-interval curve is independent of the pacing rate. As the pacing rate increases, action potential duration decreases and strength-interval curves shift to shorter intervals as shown in Figure 9.

Ranjan et al. [41, 42] suggested that the dip in the anodal strength-interval curve could be explained by the effect of a slow hyperpolarization-activated inward current. Roth and Chen [43] added such a current to the original Luo-Rudy model [44] and compared the strength-interval curves with and without this extra current. In order to determine the underlying cause of the dip in the strength-interval curve, they calculated the membrane current and the electrotonic current following the S2 shock at the location where excitation begins. They found that while both electrotonic current and a hyperpolarization-activated current contribute to anode break excitation, only the electrotonic interactions are responsible for the dip in the anodal strength-interval curve [43]. 

Joung et al. [45] proposed that the intracellular calcium concentration and sodium-calcium exchange current may play a role in determining the dip in the anodal strength-interval curve. They wrote that “anodal stimulation hyperpolarizes the cell membrane and increases the intracellular Ca^2+^ (Cai) transient. This study tested the hypothesis that the maximum slope of the Cai decline (−(dCai/dt)_max⁡_) corresponds to the timing of anodal dip on the strength-interval curve and the initiation of repetitive responses and ventricular fibrillation (VF) after a premature stimulus (S2).” Joung et al.'s paper presents an alternative mechanism that is fundamentally different than the electrotonic mechanism predicted by the bidomain model [46]. Simultaneous measurements of transmembrane potential and intracellular calcium concentration indicate that virtual electrodes can impact calcium [47, 48] but the influence of calcium on the excitation threshold remains an open question. 

Sidorov et al. [32] used a high-resolution imaging system to study the spatial and temporal stimulation patterns in Langendorff-perfused rabbit hearts. Their goal was to understand the basic mechanism of stimulation and excitability of cardiac tissue under unipolar cathodal and anodal stimulation. Particularly, they investigated the role of the virtual electrodes during cathodal and anodal excitation and tried to determine their strength-interval relationship. They found “complex dynamics” (i.e., a dip, plateau phase and a descent at the end of the relative refractory period) in the anodal strength-interval curve and a hyperbolic shape of the cathodal strength-interval curve and also observed a “damped wave” response in the dip of the SI curve [49]. Their optical mapping studies of rabbit hearts verified that the dip occurs only during break excitation [32].

Recently, Colli Franzone et al. [38] studied the cardiac excitation mechanisms, wave front dynamics, and strength-interval curves using bidomain simulations. They considered a three-dimensional cardiac slab rather than two-dimensional geometries or three-dimensional cylindrical fiber bundles that by symmetry reduce to two dimensions. The bidomain model is coupled with the Luo Rudy Dynamic (LRD) [50] model of mammalian ventricular action potential, augmented with electroporation, outward, and funny currents. They also included transmural fiber rotation and unequal orthotropic anisotropy of the conducting media. They focused on an in-depth assessment of the transition between break and make stimulation responses in the SI curves. 

**Figure 10:**
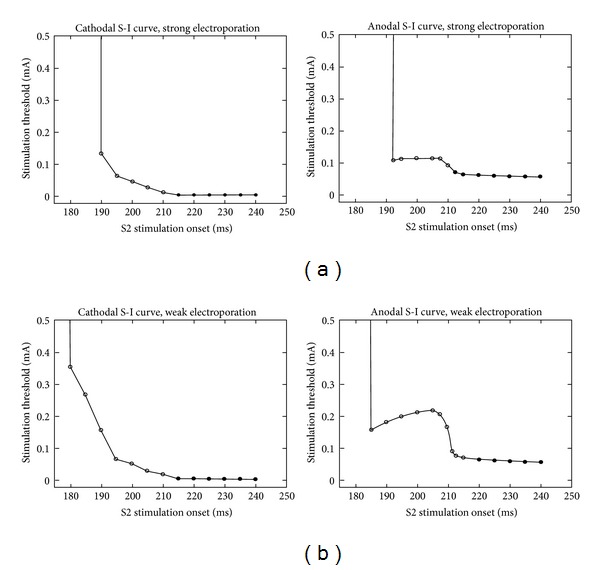
The calculated cathodal and anodal strength-interval curve for S2 pulse duration of 10 ms for strong (a) and weak (b) electroporation current. Open and filled circles denote break and make stimulation responses, respectively [49].

Tissue active response to a cathodal and anodal stimulation pulse has been studied both in terms of the time and location of the initial excitation and propagation of the wave front. In the cathodal SI curve, for intervals greater than 215 ms Colli Franzone et al. found only the cathode make mechanism with proximal excitation. For intervals between 180 and 205 ms, they found only the cathode break mechanism with distal excitation. Distal excitation response takes place at the virtual anode (located away from the electrode) and proximal excitation response takes place at the virtual cathode (located near the electrode). At the transition from break to make (intervals between 205 and 215 ms), they found “paradoxical” excitation behavior. Similarly in the anodal SI curve, they found only the anode make mechanism with proximal excitation in the interval greater than 220 ms, only the anode break mechanism with proximal excitation in intervals between 207.5 and 220 ms, and a paradoxical excitation mechanism at the transition from break to make (intervals between 207.5 and 220 ms) (Figure 10). The excitation mechanism is “paradoxical” when a nonpropagating active make response ultimately triggers excitation, in a process similar to a “damped” [49] or “graded” [51] response. The small dip in the 20 ms cathodal strength-interval curve shown in Figure 4(a) (310–320 ms) arises from a similar mechanism. Colli Franzone et al. studied this paradoxical excitation behavior in detail and found out that it is independent of (i) the electroporation current, (ii) the orthotropic or axially symmetric anisotropy, and (iii) the intramural fiber rotation. 

In the studies we have considered so far, the S1 and S2 stimuli were applied at the same location. The results may be different if S1 and S2 stimuli are applied at different locations [48, 52], a scenario often known as the “pinwheel experiment.” Lindblom et al. [53] use a two-dimensional bidomain model to examine this case. The S1 stimulus is applied at the left or bottom edge of the tissue and produces a planar wave front propagating parallel or perpendicular to the fibers. At a given S1-S2 interval, a unipolar S2 stimulus is applied through a centrally located extracellular electrode. They observed complex behavior, including both figure-of-eight and quatrefoil reentry, depending on the protocol: S1LS2C, S1TS2C, S1LS2A, and S1TS2A, where L or T correspond to longitudinal or transverse directions of the S1 wave front, and C or A correspond to a cathodal or anodal S2 stimulus. They found different strength interval curves for each of the four protocols. 

## 5. Conclusion

Predicting the strength-interval curve provides an excellent way to test mathematical models of cardiac excitation. The bidomain model predicts the abrupt transition of the anodal SI curve between make and break sections, the dip in the break section of the curve, and the relative shapes of the cathodal and anodal curves. The mechanisms responsible for the shapes of these curves, and in particular the interaction of adjacent regions of virtual electrode polarization, are the same mechanisms thought to be responsible for the induction of reentry [54, 55] and defibrillation [56, 57]. 

Over the past decade, the bidomain model has become a common tool for simulating cardiac electrophysiology [58]. Recent defibrillation studies using the bidomain model account for the heart shape and fiber geometry [59]. The simulations predict the same types of behavior discussed in this paper—including make and break excitation—but in more realistic conditions. Indeed, the phenomena responsible for the shape of the cardiac strength-interval curve are now thought to explain defibrillation success and failure.
